# Mandating Emergency Management in Nursing Homes in the V4 Countries With the Support of the Public Administration—A Mapping Study

**DOI:** 10.1155/nrp/5135506

**Published:** 2025-12-18

**Authors:** Irena Tušer, Hana Bohušová

**Affiliations:** ^1^ Department of Security Management, AMBIS University, Prague, Czech Republic; ^2^ Department of Economics, Economy and Public Administration, AMBIS University, Prague, Czech Republic

**Keywords:** contingencies, crisis management, multicriteria decision analysis, nursing homes, public administration, risk management, safety impact assessment, the elderly, vulnerable groups

## Abstract

This paper considers the assessment of the benefits and needs of the position of crisis manager in homes for the elderly (nursing homes or NH) in the countries of the Visegrad Four (V4). It is essential to reinforce safety management at these facilities in view of the increasing vulnerability of the elderly resulting from demographic changes and the increased risk of disasters caused by both natural and anthropogenic factors. The paper analyses the situation at NH in Central European countries, where the establishment of the role of the manager for emergency situations is not currently mandated, although such a position contributes significantly towards protecting the lives and health of the elderly. The methodology of the paper includes an analysis of the existing crisis management frameworks at NH and an assessment of benefits of such a role on the basis of the data obtained and comparative studies. The aim is to offer an insight into the need for the position of crisis manager in connection with increasing the safety and resilience of homes for the elderly against various types of threats and, thereby, improving their ability to respond. The results of the mapping study are universal and serve as a basis for the revision of and possible changes to national guidelines relating to crisis management at NH in both the V4 states and elsewhere.

## 1. Introduction

One of the key obligations of the state in its role in relation to public law is to ensure the protection of the lives and health of its inhabitants against various threats of both an anthropogenic and naturogenic nature [1].

A specific group that require increased attention are the elderly living in residential care facilities, also known as nursing homes (NHs) [2]. These institutions accept individuals who, in view of their advanced age, their health and their personal needs, are unable to live independently and require a higher level of care.

NH in developed countries approach the protection of vulnerable groups [3] through a set of activities that include the implementation of preventive measures, crisis planning, crisis management and risk management [4–6]. This set of activities is essential for an effective response to unexpected events such as natural disasters, pandemics, technological disasters and infrastructure failures [7, 8]. Examples might be tornadoes (in Kentucky and surrounding states in 2021; in the central and southern states of the United States in 2023), floods (in the Czech Republic, Slovakia and Austria in 2002; in England and Wales in 2007) or heatwaves (in the northwest USA and Canada in 2021; in the United Kingdom in 2022). Another example might be technological/chemical accidents (in Japan in 2011; in the city of Leverkusen, Germany in 2021; the collapse of the power grid in Texas in 2021; the SolarWinds cyber‐attack in 2020). The given examples of disasters point to the need for increasing the resilience of regional infrastructure, including NH. The resulting events emphasise the need for a specific and localised approach to planning and management that would take into account not merely general risks, but also local conditions such as demographics, geography and regional industrial distribution [5, 9].

The mapping study presented here follows on from the results and conclusions of the national research ‘Preparedness of Homes for the Elderly for Emergency Events’ [10], in which 70% of NH in the Czech Republic (370 NH) participated. The authors of the study focus, for this reason, on assessing the necessity of the position of crisis manager (CM) into the general management of NH. The aim is to support and emphasise the benefit of the mandatory inclusion of crisis management/CM in working teams at NH in order to increase the protection of the elderly in the context of the specific challenges presented by NH.

Those responsible for running NH (local authorities, churches, the private sector) in the Central European countries of the Visegrad Four (V4), i.e., the Czech Republic, the Slovak Republic, the Republic of Poland and Hungary, are not obliged by any legislative act to establish the working position of manager for emergency situations (emergency manager [EM]) to enhance safety and protect the lives and health of the elderly at NH. The reasons they give to excuse their failure to meet the obligation [1] of implementing security/crisis management include, for example, its time‐consuming nature, the complexity of maintaining security/crisis plans and the high costs involved [11]. The costs of establishing and continuously maintaining the working position of EM are higher in low‐income and middle‐income countries (e.g., the V4 countries) [12] than they can afford to cover, and the workload of an EM does not cover the full working day (1 day = 8 h). On the other hand, the working activity of a CM meets the obligation of complying with the principles of risk management in the context of the protection of the clients, employees and property of NH [13]. The incorporation of the position of EM into working teams at NH is motivated by a number of factors that emphasise its importance and necessity [6]:

1. Increasing emergency preparedness [14]: The EM ensures that homes for the elderly have prepared and updated plans for these events, including evacuation strategies and communication with the emergency services.
2. The improvement of safety documentation [9]: In addition to responding to external threats, the EM also focuses on the prevention of internal risks, such as gas leaks, electrical faults and the spread of infectious diseases. Effective protocols and regular training, including practical staff training, help reduce the risk of the inception and spread of such threats.
3. Promoting the psychological well‐being of residents [15]: The EM works with mental health professionals to ensure that the services of psychologists are available and that residents have access to information and resources that help them manage the stress and anxiety associated with emergency situations.
4. Optimising the use of material and human resources [16]: Effective resource management is crucial during a crisis. An EM ensures that necessary supplies such as medicines, food, hygiene products and other essentials are available, even if supply chains are disrupted.
5. Increasing family and community trust [17]: Systematic and effective preparation for emergencies and crises increases the trust of families who entrust their loved ones to the care of NH and improves relations with the wider community.
6. Legislative and regulatory requirements [18]: In some jurisdictions, there may be laws or regulations that require facilities for the elderly to have clearly defined procedures for the management of crises and emergencies. An EM helps ensure compliance with such regulations.

The role of the EM at NH thereby enables a comprehensive approach to risk management, increases overall safety and ensures that measures are taken to protect the elderly and the employees of NH in the event of an emergency.

For the reasons stated above, the authors of the study asked themselves the question ‘Is the effort expended on establishing and operating the working position of CM at NH appropriate in the context of augmenting the protection of clients, staff and property?’ In order to find an answer to the question defined in this way, it was necessary in the first phase of the mapping study to establish an overview of the knowledge of the current situation in relation to the given issue. In the second phase, a research investigation was conducted with representatives of local authorities and the emergency services.

### 1.1. An Overview of Knowledge of the Current State of Crisis Management at NH

The overview of knowledge of the current state was elaborated in four areas:

1. First and foremost, in the area of the legislative acts of the V4 states, quality standards and planning documentation covering safety at NH;
2. questions of the value of human life;
3. a search of the literature—academic studies;
4. methods for decision‐making.

#### 1.1.1. An Overview of Knowledge of Legal Standards, Quality Standards and Planning Documentation at NH in V4 Countries

The V4 states, which have many features in common, represent a platform for co‐operation and coordination in political, economic and security areas and share similar historical experiences, were selected for the overview of knowledge in the first area.

The legislative acts of the V4 states respect the principles of democracy and constitutionality, including the Charter of Fundamental Rights and Freedoms [1]. One of the fundamental obligations of democratic states is to ensure the protection of the health and life of their population, including the elderly.

The protection of the population is defined by a number of laws in the Czech Republic [19] (Act No. 110/1998 Sb. on the Security of the Czech Republic [20]; Act No. 239/2000 Sb. on the Integrated Rescue System), though these are not, however, linked to the laws regulating the operation of NH [21] (Act No. 108/2006 Sb. on Social Services [22]; Decree No. 505/2006 Sb. implementing certain provisions of the Social Services Act). The laws regulating the principles of operation of NH do not define the principles of crisis management (emergency management) [10]. This leads to the fact that crisis management at NH is not addressed in a comprehensive manner, and those responsible for these facilities do not have clearly defined responsibilities or support in the area of EM. This situation results in limited (or no) engagement of professional EM and limited protection of vulnerable groups of the population.

The fundamental idea behind the state administration of the Czech Republic is security, and the EM for vulnerable groups of the population is ensured by external entities (bodies of the state and local administration for crisis management and teams of the integrated rescue system). The contribution towards the EM made by those responsible for NH is not covered in a comprehensive manner by the pertinent laws or supported financially by the state administration or local authorities. This is one of the principal reasons why the operators of NH, who are responsible for the safety and operability of residential facilities, do not respect the importance of the working position of CM (EM). The protection of the clients, employees and property of NH is realised in individual sections and areas. This involves the areas of health and safety at work, fire protection and first aid, with an evacuation plan, a pandemic plan and a plan for operational emergency situations (e.g., burst water pipes, short‐term interruptions to the electricity supply and malfunctions to elevators) being elaborated in the planning documentation.

A similar attitude can be identified at NH in the Republic of Poland. The operation of and conditions in homes for the elderly in Poland are regulated principally by the Act on Social Services No. 194/2004 [23]. This law sets out basic standards and requirements for facilities providing 24/7 care to the elderly, the disabled and the chronically ill. The EM in NH is not specifically regulated by any special law but is implicitly covered by the broader health and safety provisions that comprise part of this act. The responsibility for the possible implementation of crisis management rests with local administrative authorities and facility operators who adopt measures in the event of any emergency to the best of their knowledge and abilities. This situation subsequently leads to variability in the implementation of crisis measures.

The basic legal framework for the operation of homes for the elderly in Hungary is also established by the law [24]. This law contains basic regulations for the operation of social facilities, including homes for the elderly, and ensures that these facilities meet certain standards of care and safety, as is the case in the other V4 countries. Specific requirements for crisis management are elaborated in Decree No. 1/2000 of the Minister of Social and Family Affairs [25, 26], which sets out standards of care and includes measures for the management of crisis situations such as pandemics and natural disasters. This decree requires each facility to have an emergency plan in place that takes in staff training and the assurance of resources necessary for emergency situations. This concept is not, however, comprehensive. An analysis of the given sources reveals the absence of the principles of risk management, methodical guidance for the elaboration of plans of preparedness and practical training of the necessary skills.

The government of the Slovak Republic recognised the necessity of crisis management in residential facilities for the elderly [27] in response to the experience gained in handling the COVID‐19 pandemic. This led to the consideration of the problem of how to mandate the obligation of crisis management centrally on all operators of NH and augment their crisis preparedness for potential threats in a continuous manner. The Slovak government handled the situation that had arisen by approving a legislative act on the implementation of certain measures (e.g., crisis planning and crisis management) in accordance with a system of economic mobilisation [28]. It classified all NH among entities of economic mobilisation. The Slovak government stipulated the legal obligation for all operators of NH to apply the principles of crisis management and the obligation of elaborating a crisis preparedness plan for handling crisis situations associated with the pandemic [29] (Table 1). The approach taken by the state authorities in Slovakia is unique and underlines the efforts of the state administration to manage security in relation to seniors.

**Table 1:** Overview of knowledge in V4 countries.

Documentation analysed	Title	CR	SR	POL	HU
Legislative acts for NH	Covering individual aspects of safety (health and safety at work, fire protection, first aid)	✔	✔	✔	✔
	In part covering crisis management	X	✔	X	✔

Quality standards at NH	Covering individual aspects of safety (hygiene, diet, environment, culture, activities)	✔	✔	✔	✔
	In part covering crisis management	X	X	X	X

Internal planning documentation	Covering individual aspects of safety (health and safety at work, fire protection, first aid, emergencies in buildings)	✔	✔	✔	✔
	In part covering crisis management	X	✔	X	✔

*Note:* Source: elaborated by the authors.

The overview of knowledge in the field of crisis management under the conditions in force at NH in the V4 countries demonstrates the absence of a comprehensive approach (binding work guidelines, instructions for the performance of tasks and the definition of procedures for all types of threats/risks). The Czech Republic and Poland show deficiencies in legislative support for crisis management at NH. Hungary and Slovakia have more sophisticated systems anchored in the legislation, while Slovakia has implemented an innovative approach that primarily reflects the threat of infectious diseases (pandemics).

A brief overview of the results of the analysis of the application of crisis management at NH in the V4 countries is presented in Table 1.

### 1.2. The Value of Human Life

When assessing the benefit and necessity of the (to date nonexistent) position of the CM at NH, it is also important to express an opinion on the ethical question of the value of the human life of an elderly person at an NH.

Determining the value of human life (an NH client and an NH employee) can be understood from two points of view. From an ethical and a moral point of view, human life cannot be expressed in monetary units, because life is the highest value and the right to life is inalienable. From an economic point of view, the quantification of the value of life in monetary terms is taken into consideration for the purposes of insurance or litigation (compensation, reparation, indemnification). The criteria of the individual’s age and economic activities within the framework of their profession are taken into consideration during the determination of judicial compensation [30]. A person also creates other values as well, however, over and above their professional occupation. The impact on the relatives of the deceased individual and their state of mind, which cannot be measured with any precision, are also taken into consideration. A person’s overall lifelong contribution to loved ones, family and society is taken into account. The way in which the value of life is quantified in monetary terms differs in individual countries depending on economic, social and cultural factors [30].

When we talk about economic value, experts generally use the concept of the Value of a Statistical Life (VSL), which is used to estimate how much money society is willing to expend to reduce the risk of death [30, 31]. The VSL is generally used to assess risk and policies in health, transport and the environment and estimates how much money society is willing to pay to reduce the risk of death [32].

### 1.3. Literary Research of Expert Studies

Most of the research studies in this area focus on primary research at long‐term care facilities for the elderly. A total of 6550 records were identified in the Web of Science database of scientific studies using the keywords long‐term care facilities or NH and safety in the title. These studies consider various aspects of patient safety, shedding light on critical factors influencing safety climates, the reporting of adverse events and the challenges faced by the managers and staff of healthcare facilities in the provision of high‐quality care.

A significant proportion of studies in the area of client safety at long‐term residential facilities (5621) focus on developed countries such as South Korea, Norway, the United States, Canada, Australia, the Netherlands, Singapore, Germany, Great Britain, Italy, Norway and China. A total of 130 studies are devoted to V4 countries, of which 90 relate to Poland, 21 relate to the Czech Republic, nine relate to Slovakia and ten relate to Hungary.

Significant attention and extensive research are devoted to ensuring the safety of clients at long‐Term Care Facilities (LTCF) (equivalent to NH) in South Korea. The authors Min et al. consider the safety of clients at LTCF in their studies both from a practical viewpoint [2] and from the viewpoint of analysis of research already conducted in this area [33]. Min and Yu [33] shed light on the diverse safety challenges at Korean LTCF, taking in the administration of drugs, the control of infection and fire prevention. The conclusions of this study emphasise the complex nature of the situations that may threaten the health or life of clients at LTCF, which demands a comprehensive approach to, first and foremost, the minimisation of risk.

Kim et al. [34] also underline the importance of organisational factors in shaping the culture of patient safety at homes for the elderly. Their review highlights the key role played by staff education and staff composition in promoting an environment favourable to patient safety. Choi et al. [35] emphasise the increased attention devoted to the safety of people at LTCF, primarily in view of the greater risk to which they are exposed in emergency situations due to their age, limited mobility, illnesses and mental state. They propose a special educational programme with the aim of improving the management of emergency situations and interprofessional co‐operation. Choi and Chang [36] address problems in communication during emergency situations at homes for the elderly.

Another country that places an emphasis on assuring the safety of clients at LTCF (it refers to them as NH) is Norway. The research team (Wiig, Aase, Johannessen, Holen‐Rabbersvik, van de Bovenkamp, Bal and Ree) [37] addressed the quality of care and client safety in their research project at the University of Stavanger. The aim of their work was to develop a tool for mapping the quality and safety context adapted to the NH and homecare environment.

The authors led by Ree and Wing [38] examine the perception of the culture of patient safety at Norwegian NH and homes for the elderly from the perspective of their employees. Building an effective team workplace in homecare and promoting open communication at NH are appearing as key strategies for improving the culture of patient safety.

According to Mageroy [39], managerial staff in healthcare are bearing increasing responsibility for the performance of employees, patient experience and safety and the quality of the care provided. The aim of this research was to investigate how managers of care institutions manage the dual responsibility of both Health, Safety and Environment (HSE) and Quality and Patient Safety (QPS). The study showed that managers of care institutions who are responsible for ensuring quality and safety for the patients and staff of these facilities experience the stress of managing this dual responsibility. They recognised the importance of having time to be present as a manager, having robust systems for maintaining HSE and QPS, and the fact that conflicting aspects of the legislation are a daily challenge. Their conclusions highlight the need for robust support systems to maintain the standards of care and safety for both patients and employees in the event of an emergency or crisis situation arising.

Other studies in the area of safety present the views of patient safety and quality of care from various perspectives. The factors that influence the level of safety are considered by Świtalski et al. [40] in their study of secondary sources. The aim of this international study of publications focussing on the safety of clients at long‐term care facilities is to identify interventions that may contribute towards increasing patient safety at long‐term care facilities. The authors identify factors such as understaffing, unsuitable facilities and insufficient funding and regulation as key factors increasing the risk of the adverse impact of crisis events. The conclusions of this study of secondary sources emphasise the necessity of facilities following the guidelines drawn up by institutions engaged in patient safety.

Kiljunen et al. [41] present a qualitative study concerning the perception of barriers and contributing factors in the daily work associated with the safety of residents and patients at residential facilities and homes for the elderly. It identifies the following factors: (1) competent personnel and material resources; (2) management and culture; (3) communication, networks and optimal use of expertise; and (4) effective application of guidelines, rules and regulations that play a significant role in client safety. Their conclusions emphasise the importance of competent staff, effective management and interdisciplinary co‐operation in promoting the safety of residents and patients at NH.

Li et al. [42] and Quach et al. [43] examine the link between the safety climate and organisational preparedness for change at a facility for the elderly in the United States, emphasising the need for proactive initiatives to improve resident safety. They state the creation of a culture of patient safety and organisational preparedness for changes in this area as possibilities, giving examples—team meetings and organisational initiatives represent the opportunities for recognising and applying the accumulated safety knowledge of experienced members of staff.

Attention is also paid to unexpected risk events such as fires, floods and earthquakes. The authors of [44] consider it is important that the staff of NH is equipped with proper concepts of disaster prevention, responses to emergency survival and measures mitigating danger. The aim of their study was to analyse the effectiveness of interventions, various kinds of training for fire prevention and responses to emergencies at NH. This study found that the key to improving the effectiveness of learning involves the addition of a chapter on fire science when drawing up fire safety training materials in order to reinforce basic awareness.

The conclusions of the majority of studies indicate that, in addition to assuring the safety of clients in the area of mandatory measures related to property, high‐quality staff with adequate knowledge and expert healthcare, it is also necessary to create a high‐quality system with a view to crisis management, including the identification of external and internal risks and their control under the conditions in force at the given residential facilities for the elderly.

### 1.4. An Overview of Knowledge of Methods for Decision‐Making

The context of the decision (whether to incorporate a CM into the management of an NH or not), the characteristics of the given plan and the available information were considered during the selection of a method for the subsequent research investigation. Each decision‐making method has its own specific features that must be considered before its application. For the given reasons, the authors of the study first identified applicable methods and identified the positive and negative aspects (Table 2) of them that they considered applying. These methods were cost–benefit analysis (CBA), cost‐effectiveness analysis (CEA), multicriteria decision analysis (MCDA) and risk–benefit analysis (RBA).

**Table 2:** Identification of the basic specifics of each decision‐making method.

Method	Positive and negative	Specifics of the method	Studies carried out
CBA	(+)	Quantification of benefits and costs Transparency Flexibility	Edwards and Lawrence [45]
			Tessier et al. [46]
			De Gruyter et al. [47]
			Mann et al. [48]
			Battistoni et al. [49]
	(−)	The monetisation of benefits The underestimation of long‐term Benefits or costs Distribution effects	Rahja et al. [50]
			Kok et al. [51]
			Glied and Robinson [52]
			Lamfre et al. [53]

CEA	(+)	Focused on results Comparison of alternative interventions For decisions on treatment methods	Isaranuwatchai et al. [54]
			Smith et al. [55]
			Penkunas et al. [56]
			Hoedemakers et al. [57]
	(−)	Projects with multilateral goals Considering only the effectiveness of costs Lack of flexibility	Kleijburg et al. [58]
			Michelly Gonçalves Brandão et al. [59]
			Vestjens et al. [60]

MCDA	(+)	Considering multiple factors Support for dynamic decision‐making Support for a consensus	Saarikoski et al. [61]
			Karim et al. [62]
			Islam et al. [63]
			Rutten‐van Mölken et al. [64]
	(−)	Subjectivity Time‐consuming and complicated	Hoedemakers et al. [57]
			DiStefano and Krubiner [65]
		Inadequate transparency	van den Bogaart et al. [66]

RBA	(+)	Risk assessment Balanced decision‐making Planning	Schnarr and Mertz [67]
			Huang et al. [68]
			Thomsen et al. [69]
			Guo. et al. [70]
	(−)	Unavoidable uncertainty Complexity of assessment Irrational preferences	

*Note:* Source: prepared by the authors.

Although the CBA method is used in a number of studies for the purposes of evaluation and decision‐making in healthcare and social care, for example Edwards and Lawrence [45, 71] and Mann et al. [48], the authors chose the MCDA method for further processing [12] on the basis of their assessment of the basic specifics, positives and negatives of the given methods. The use of the given method is based on the basic mission of NH, which is not to make a profit, their goals being focused primarily on the provision of a safe and high‐quality environment for the elderly that should enable them to live a dignified and satisfied life in their own apartment or room with the necessary level of assistance and ensure them high‐quality care, including assistance with personal hygiene, dressing, eating, administering medication and other daily tasks.

The use of MCDA in health and social research continues to grow, in part, on the basis of the results of the theoretical study by Gongora‐Salazar et al. [72], with the majority of such studies (49%) serving for decision‐making in relation to the determination of priorities. The most commonly used criteria are the safety, cost and quality of the provision of care, although there is a considerable variation in different decision‐making contexts. The MCDA method is suitable for support in the case of problems in which there may be conflicting economic, environmental, social, institutional, technical and aesthetic goals. Marsh et al. [73] are important proponents of the given method, particularly in healthcare. This method is used for the purposes of decision‐making in the field of social services by, for example, Karim et al. [62] for the evaluation of a comprehensive programme of healthcare and social care and Hoedemakers et al. [57], who point out the shortcomings of methods focussing exclusively on the analysis of cost‐effectiveness, since they may fail to record the value created by innovations in care for the elderly.

Decision‐makers in healthcare and social care face the global challenge of developing reliable evidence‐based methods for deciding what should be funded. Allocating limited resources across different needs is difficult, because a number of criteria other than simply economic criteria are important to healthcare and social care. When comparing options in healthcare and social care, decision‐making bodies (those operating facilities = local authorities, churches and the private sector) often look for compromises between these criteria. MCDA is a tool that helps decision‐makers summarise complex value compromises. The criteria that are taken into account when determining priorities can make it clear to the parties involved how responsible authorities make decisions [74]. The goal of those making decisions is to make the best choice among alternative courses of action that are characterised by multiple criteria [75]. Such a procedure can help ensure that the decisions made are consistent with the stated objectives of the entity concerned.

## 2. Materials and Methods

The precondition to the use of MCDA is that the set of evaluated variants is described explicitly by the specific enumeration of all elements. The essence of the method lies in the determination of criteria, i.e., the aspects for evaluation. These criteria can be expressed as a numerical value—quantitative and qualitative (in this case, the qualitative evaluation must be quantified into a selected scale of values). The decision criteria are established with a view to the goals of the decision‐makers. It follows from this that decision‐makers may have different values with different sets of goals and preferences. For the purpose of making a final decision, it is appropriate to modify the multicriteria model of decision theory into a single‐criterion model.

The additive or weighted sum method [75] using formula (1) is a widely used method of aggregating the preferences of decision‐makers,

(1) Va=w1×s1a+w2×s2a+…+w10×s10a,Va=∑i=1nwi×sia,

where

– *w*_*i*_ is the weight (importance) of criterion *i*,
– *s*_*i*_(*a*) is the score of alternative *a* for criterion *i*,
– *V*(*a*) is the overall aggregated score of alternative *a.*

*v*_*i*_(*a*) is the evaluation of the programme alternative in the *i*th criterion [75, 76]. The partial value functions *v*_*i*_ are bound by 0 (the worst result) and the best result (e.g., 1). They can be assessed using a variety of techniques, including the use of a direct rating scale [77]. The importance of criteria *i* is represented by swing weights, where the weight *wi* represents the scale and relative importance of the *i*th criterion [75, 78, 79].

Objectives in healthcare and social care can be divided into three groups of interested parties [80]:

1. Goals for the client (a resident of an NH)—criteria that contribute to the value of the social care that the NH provides (feeling of safety, assuring the continuity of care even in the event of emergencies, prevention of health problems and compliance with quality standards).
2. Goals from the viewpoint of society (increasing or assuring safety for a large group of people, assuring the protection of property, benefiting a large group of people and focussing on vulnerable people).
3. Sustainability (economic impacts, effectiveness and demands on human resources).

**Figure 1:**
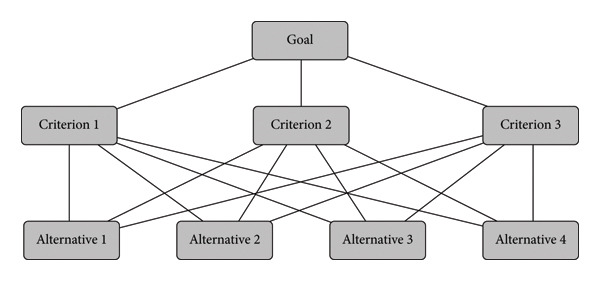
The basic structure of the analytic hierarchy process method (without subcriteria) [12].

Since the medical literature provides few leads for the selection of criteria and weighting methods, the authors base their determination of criteria on the aforementioned goals of healthcare and social facilities, and the selection of scoring and weighting techniques also follows from this. These are based primarily on Marsh et al. [73] who consider scoring and weighting techniques used in the health and social sectors. The objective of the weighting and scoring steps in MCDA is to capture the priorities and preferences of interested parties for individual criteria to assess the relative overall value of the options (see Figure 1).

‘Direct assessment’, which represents the simplest set of available methods for estimating the weights of criteria (interested parties assign numerical values to individual criteria without the occurrence of compromise), was used in the mapping study implemented. Determining the levels of criteria on the basis of which alternatives are scored according to a given criterion is a type of ‘scoring function’.

Evaluation criteria were created in connection with the stipulated goal of the mapping study to substantiate the need for the introduction of the position of CM into the management of NH:

1. The client: safety and protection, continuity of care and reduction of stress and anxiety
2. Society: safety of people, protection of property, improvement of communication, rapid response to emergency events, satisfaction of employees and others
3. Sustainability: costs and savings, reputation and credibility

Research aimed at assessing the necessity of the position of CM was conducted at local government public institutions in all the regions of the Czech Republic (14 regions) and with the emergency services of the Czech Republic. Key criteria were first identified on the basis of previous studies in the field of healthcare and social facilities (Table 3). The weight of the criteria was determined on a scale of 1–10 from the results of the literature survey, while the impact of the introduction of the position of CM was evaluated on a scale of 1–5. Before the introduction of the given position, the impact value for each criterion was set to 1, which represents the fulfilment of basic requirements. When the values (weights and impact) were assessed by the group of evaluators, the weight of individual criteria was determined, and the impact of the introduction of the position of CM on these criteria was analysed. For the purposes of evaluation, the average values of the assigned weights of criteria and impacts, as determined by the research survey, were used (Table 3).

**Table 3:** Assessment of criteria on the incorporation of a crisis manager into the general management of NH.

**Area**	**Main criteria**	**Significance** **w**_**i**_	**Effect** **v**_**i**_

Client	1. Improving standards of quality	4.4	2	
	2. Increasing protection of lives and health during natural and anthropogenic threats	8.27	4.57	
	3. Continuity of care measures during the resolution of threats/emergencies	6.4	2.86	
	4. Reducing stress and anxiety by means of the application of the principles of crisis communication during the resolution of emergencies on the part of NH staff	7.07	3.36	

Society	5. Support for faster responses by the management of NH to any emergency arising	8.47	4.14	
	6. Increased protection of the property of NH (movable and immovable property)	7.13	3.43	
	7. Greater reputation and credibility	5.13	2.28	

Sustainability	8. Costs of introducing the position of crisis manager	7.13	3.86	
	9. Costs of maintaining the position of crisis manager	6.93	−3.79	
	10. Savings associated with the resolution of emergency events and the restoration of operations (the crisis manager will introduce standards of crisis management, thereby reducing the negative impact of emergencies on the operability of the facility)	7.80	3.71	

The group of evaluators (respondents) are experts linked professionally with crisis management at public institutions (authorities) responsible for the safety and protection of the population within the national economy. The given authorities are established by the regional government. Local government authorities are also operators of NH in individual regions and are therefore jointly responsible for the protection of the clients of NH. Persons with nationwide responsibility for the safety and protection of the population (from the Police of the Czech Republic, the Police Presidium and the General Directorate of the Fire Rescue Service) also participated in the research investigation. A total of 32 respondents (the group of evaluators) took part in the survey in the period from April to June 2024.

One limitation of the mapping study is the restricted possibility of generalising its conclusions due to the fact that the sample of participants came exclusively from public local government institutions and the emergency services of the Czech Republic. The authors believe, nevertheless, that the results of the research investigation and the findings from the literature research and discussion contribute sufficiently to their final statements, which can be generalised both for other states and for a number of ministerial departments (education and healthcare).

## 3. Results

### 3.1. Findings

It was found from the overview of knowledge of the current situation relating to the issue under consideration here that crisis management is implemented in developed countries under the conditions in force at NH (see the chapter Literary Research of Professional Studies). The given problem is considered only in part in the V4 countries. There is no mandatory regulation that would stipulate the obligation of applying risk management in a comprehensive manner at NH, thereby increasing the safety/protection of the facility. There is no departmental methodical management or implementation of education and training in the area of EM modified for the conditions in force at residential facilities for the elderly. This gives rise to the heterogeneity of crisis preparedness at the given entities.

The counterarguments of the public administration and operators of NH are generally based on the costs and the time‐consuming and complex nature of maintaining crisis plans. For this reason, a research question was posed in the introduction to the mapping study: ‘Is the effort expended on establishing and operating the new working position of CM at NH appropriate in the context of increasing the safety of clients and staff?’ The answer YES, based on the argumentation, results from the literature research and from the results obtained by the research investigation among representatives of regional government and the emergency services in the Czech Republic.

The values obtained from the research aimed at assessing the necessity of the position of CM into the general management of NH are presented in Table 3.

When using MCDA, a decision can be made based on the above set of criteria regardless of other criteria. When this method is used to adopt a decision on the introduction of the position of CM, two alternatives that differ in the criteria listed in Table 3 are considered. It is assumed that the other criteria that are not listed in Table 3 are the same for both alternatives.

The main resultant indicator in the MCDA was the total value score calculated as a weighted sum of the ten resultant criteria. These criteria included the areas of the client, society and sustainability of crisis management. Variants were subsequently compared from the data obtained with the application of formula (1). A higher value means greater importance to the operator of the NH.

Variant 1: the existing situation without a CM,

(2) Va=∑i=1mviwi a=26.42.

Variant 2: with the position of CM,

(3) Va=∑i=1mviwi a=190.07.

As is evident from the resultant values for each of the variants *V*(*a*), the value for variant 2, i.e., for the introduction of the position of CM, is significantly higher (190.07) as compared to Variant 1 without a CM (26.42). This assessment reflects the attitude of the group of evaluators. The authors endeavoured to eliminate their own subjectivity in this way.

Following up from the confirmation of the importance of the incorporation of a CM into the working team at an NH, a methodological procedure entitled Preparation of a Plan of Preparedness for Residential Social Service Facilities to Prepare Preventive Measures for Emergencies was elaborated [10, 81]. This methodological procedure presents the guideline recommendations for the implementation of individual steps respecting the principles of crisis management. Its application under the conditions in force at NH has been approved by the Ministry of Labour and Social Affairs and the Association of Providers of Social Services of the Czech Republic [82]. This methodological procedure is currently being considered by the Government Council for Seniors and Population Ageing. On the basis of a statement made by the Government Council for Seniors and Ageing, a mandatory approach defined by the state may be implemented for the management of safety and crisis situations in the NH environment through a CM. The adoption of the pertinent proposal by the political sphere will strengthen the resilience of NH in modern society.

## 4. Discussion

A comprehensive approach to reinforcing safety in continual care facilities such as NH requires not only a mastery of the traditional aspects of safety and crisis management that are normally addressed within healthcare but also the effective management of other less obvious risks [41, 44, 83]. Risks may be of both an external and internal nature, and both groups may have a significant impact on the safety of the clients, employees and property of these facilities [15, 84]. The given facts justify the entities responsible in implementing the corresponding preventive measures to protect this vulnerable group [44, 85].

Preparing for identified risks begins with planning tools that are part of broader crisis management and risk management strategies [11]. As a rule, these strategies are designed, planned and managed by a CM as a regular part of general NH management in developed countries [33, 38, 83]. This practice is not the rule, however, in the post‐Communist V4 states, and this poses a significant risk to the safety of the clients of the given facilities.

Planning and organisational processes intended to handle emergency events are developed by the education and training of staff through a CM [6, 35, 43, 86]. Education and training also take in communication skills in relation to the media and the public, which are critical skills for minimising panic and ensuring the proper flow of information during an emergency [36, 87, 88]. In comparison with the given facts, the operators and management of NH in the countries of Central Europe (V4) place the emphasis on ensuring safety only in the individual areas that are mandatory. These are the areas of health and safety at work, fire prevention, first aid, evacuation plans and pandemic plans. The staff at NH is educated, but not trained, in the given areas, and practical exercises/training of procedures and processes are not implemented [85, 89, 90].

Serious shortcomings may arise if the obligation of applying the principles of crisis management and risk management is not stipulated by a legal norm and continues to be left on a voluntary basis [8, 90]. The voluntary fulfilment of obligations can be unpredictable and heterogenous and tends towards irregular crisis preparedness. Differing levels of knowledge, skills and motivation among the management of NH in the implementation of crisis strategies can lead to uneven levels of crisis preparedness. In the event of a crisis situation, this may represent a serious risk not only for the residents of these facilities but also for society as a whole [89, 90].

The authors of this paper have, for this reason, focused on obtaining evidence and arguments that support the obligation of implementing crisis management strategies at NH by means of legislative acts. A mandatory regulation has the potential to influence the protection of the elderly in V4 countries and increase the level of crisis preparedness at these facilities in a positive manner [91].

Although the incorporation of a CM into the management of NH requires additional expenditures [52, 66, 92], our research study suggests that this should be perceived as participation in increasing overall safety and quality of care for the elderly. The operators of NH are, however, often reluctant to incur these costs, primarily due to their limited budgets and a lack of convincing evidence of the direct benefits of this investment [8, 83]. In contrast to this, the results of our study support the argument that the presence of a CM in the team not only contributes to the improved management of crisis situations but also increases the trust in the care provided among clients and their families, and this contributes to a better reputation and long‐term sustainability for NH. This claim is also backed up by international expert studies [83, 87, 91].

The conducted study represents an contribution to the internationalisation of crisis management research within the social care sector. Its principal strengths lie in methodological innovation, interdisciplinary integration and a direct impact on policy development.

The results primarily reflect the institutional and economic dimensions of crisis management, which are expressed in a quantitative framework. Consequently, ethical and cultural considerations regarding the value of life among older adults are discussed predominantly at the theoretical rather than empirical level. From this perspective, future research could benefit from the application of qualitative approaches to gain a deeper understanding of the impacts on clients.

Although the MCDA methodological approach enabled a systematic quantification of the benefits and costs associated with the CM position in NH, the results may be partly influenced by the subjective preferences of the evaluators.

While most international studies focus on healthcare institutions, this study extends the framework of crisis preparedness to the field of social services, where client vulnerability is particularly high. The research is applied and policy‐relevant, providing an evidence base for legislative adjustments and methodological guidance in the Visegrad Group (V4) countries.

It is necessary for the operators of homes for the elderly and the pertinent political entities to focus on re‐evaluating the approach taken to funding safety at these facilities, taking into account the importance of crisis management as a key element in the protection of one of the most vulnerable groups of the population.

## 5. Conclusion

The conclusion of this study underlines the urgent necessity of prioritising crisis management at NH. Despite the historically lower attention paid to safety at NH as compared to hospitals, this issue encompasses a wide range of aspects including the preparation of preventive measures, mobility, adaptation of the environment, assuring staffing and crisis communication training. The mapping study produced from the results obtained emphasises the necessity of a comprehensive safety framework that includes the control of both external and internal threats and that is adapted to the capacity and location of individual facilities.

A key aspect of the safety infrastructure at NH is the integration of the role of CM, which is common in developed countries but less widespread in the V4 states. The study recommends the incorporation of a CM in order to standardise safety and crisis management in accordance with the national policies of the V4 states. A methodology has been developed to support the given initiative [10]. A mandatory approach to the handling of emergency and crisis events at NH is supported by the pertinent ministry [81]. The aim of legislative support is to harmonise crisis management at residential facilities for the elderly and thereby reinforce their resilience and ensure a higher level of care for the elderly population in the context of modern society and not merely in terms of health.

This study was conducted on the basis of co‐operation between university researchers and the Ministry of Labour and Social Affairs. Based on the knowledge obtained from the mapping study, the given ministry has supported the implementation of crisis management principles under the conditions in force at NH. The academic community has contributed in this way to the improvement of practice in the given area.

## Conflicts of Interest

The authors declare no conflicts of interest.

## Funding

The authors have nothing to report.

## Data Availability

The data that support the findings of this study are available on request from the corresponding author. The data are not publicly available due to privacy or ethical restrictions.
